# Spanish validation of the simple lifestyle indicator questionnaire: validity and reliability analysis

**DOI:** 10.3389/fpubh.2023.1146010

**Published:** 2024-01-09

**Authors:** Elisabet Montero-Sandiego, Nicolás Ruiz-Robledillo, Rosario Ferrer-Cascales, Violeta Clement-Carbonell, Cristian Alcocer-Bruno, Natalia Albaladejo-Blázquez

**Affiliations:** Department of Health Psychology, Faculty of Health Science, University of Alicante, Alicante, Spain

**Keywords:** lifestyle, simple lifestyle indicator questionnaire, validity, reliability, test–retest

## Abstract

**Introduction:**

It has been shown that lifestyle is a highly modifiable determinant having a direct effect on the health status. Therefore, short and simple questionnaires assessing the lifestyle of the general and clinical population are needed to create interventions on behavioral aspects that can improve the health status. The Simple Lifestyle Indicator Questionnaire (SLIQ) is a validated health scale in English that combines five lifestyle factors: diet, exercise, alcohol consumption, tobacco use, and stress level. The objective of this study was to validate the SLIQ questionnaire in Spanish by analyzing the scale’s validity and reliability. Its discriminatory power of the scale was also examined by evaluating the differences in health outcomes according to the levels of adherence to a healthy lifestyle.

**Methods:**

The sample consisted of 745 participants with an average age of 39.94 (SD: 16.99). A transcultural adaptation process was carried out to validate the SLIQ questionnaire in the Spanish context, to determinate the structural equivalence of the Spanish version as compared to the English version, and to assess the psychometric properties of the scale. PREDIMED and IPAQ scales were used to analyze the convergent validity of the Spanish version of the SLIQ regarding to diet and exercise, and the questionnaires SF-12 and DASS-21 questionnaires were used to assess the capacity of the Spanish version of the SLIQ to discriminate health status related to different levels of reported lifestyles.

**Results:**

Regarding validity, the results indicate significant correlations between the different dimensions of the SLIQ questionnaire and those used as a reference. As for reliability, the test–retest analyses reveal a high temporal consistency for the scores obtained on the questionnaire. Finally, the differences found in anxiety, depression, and quality of life, with regard to the different levels of adherence in the SLIQ questionnaire, suggest that the questionnaire’s Spanish version has adequate discriminatory power.

**Discussion:**

The obtained correlation coefficients between the SLIQ and the other standardized measures pointed out the adequate convergent validity of the instrument. Moreover, the test–retest results demonstrated the stability of the results obtained through this questionnaire. Finally, the lifestyle categories derived from the SLIQ showed a high ability to discriminate between participants’ health profiles. Hence, it can be concluded that the Spanish version of the SLIQ questionnaire is a valid and reliable tool for the quick and effective assessment of lifestyle.

## 1 Introduction

Lifestyle (LS) is a concept referring to a person’s behaviors, from both an individual point of view and in terms of group relationships, built around a series of common behavioral patterns (1). These habitual patterns of behavior characterize a person’s way of life and may affect their health to a greater or lesser extent (2, 3). According to the World Health Organization (WHO), these behavioral patterns are constantly being interpreted and tested in different social situations and, therefore, they are not constant, but rather, they are subject to change (4). LS includes behaviors such as eating, exercising, resting, sleeping, playing, socializing, and working, as well as other aspects; in other words, it refers to an individual’s way of living (3, 5–7). The specific characteristics of LS may change over an individual’s life, although certain habits or customs may remain the same over time (5, 8, 9). It has frequently been shown that a significant relationship exists between LS, health, and quality of life (10–13).

Different studies have revealed that an unhealthy LS characterized by the use of tobacco, alcohol, and drugs, a lack of physical exercise, and unhealthy eating habits, is related to a series of pathologies such as dementia, cancer, type 2 diabetes, metabolic syndrome, and cardiovascular problems, among others (14–16). Numerous studies have found that a healthy LS is associated with a lower risk of cancer, cardiovascular disease, diabetes, and mortality, leading to a longer life expectancy and a more years of life free of these diseases (17–22). These findings suggest that the promotion of a healthy LS could help ease the burden of medical care by reducing the risk of developing multiple chronic diseases and increasing disease-free life expectancy. Therefore, to ensure a longer and better quality life, measures promoting an active and healthy LS should be implemented, paying special attention to the adoption of habits fostering quality of life (23–26).

Despite the abundance of scientific evidence revealing that maintaining a healthy LS is one of the main conditioning factors affecting health status, few assessment tools permit the quick and effective identification of LS and also assess the main LS dimensions that have been associated with a decline in health status. However, there are numerous questionnaires that allow for individualized assessment of the risk factors related to LS (1, 27–31). However, these questionnaires may be timely to complete and they may require a lot of very detailed information. This is a significant limitation both in terms of human and time resources with respect to their systematic use, especially in the field of healthcare (27).

Therefore, short and easy questionnaires that assess the LS of the general population and that may also be applied to the clinical and research field are necessary (27, 28). The Simple Lifestyle Indicator Questionnaire (SLIQ) is a validated English language health scale consisting of 12 items that assess five factors of LS: diet, exercise, alcohol consumption, tobacco use, and stress level. Moreover, the scale provides a global score for LS and a score for each of its components and is quick to complete (27, 28). The original English version of this questionnaire displayed adequate psychometric properties in terms of validity and reliability (6, 27).

Different studies have used this tool to analyze the relationship between LS and different variables, including the recent study by Znazen et al. (32) relating LS and stress induced by home confinement during the COVID-19 pandemic. Other studies have also used the SLIQ questionnaire to analyze the association between adherence to an unhealthy LS and an increase in chronic diseases, especially cardiovascular ones (16, 25, 33–35), or between the adoption of inadequate LS patterns and higher levels of stress, anxiety, and depression (36, 37).

Considering the advantages of this questionnaire ease of application and good psychometric properties, the objective of this study is to validate the SLIQ questionnaire in Spanish and analyze its psychometric properties, focusing specifically on reliability and validity. Moreover, the study also assesses the questionnaire’s ability to discriminate between health results based on the cut-off points established in the questionnaire to determine adherence to a healthy, intermediate, and unhealthy LS.

## 2 Materials and methods

### 2.1 Design

This study is part of a large-scale cross-sectional study analyzing LS in the general population and its consequences on health and quality of life. An instrumental study of the validation of the SLIQ questionnaire in the Spanish context was carried out (38, 39). Adaption of the SLIQ instrument to the Spanish context was performed in two phases. Firstly, in phase 1, a standard translation and back-translation process was carried out for linguistic validation. Then, in phase 2, the psychometric properties of the Spanish language instrument were evaluated, assessing both validity and reliability as well as the tool’s discriminatory power regarding health outcomes, specifically anxiety, depression, and health-related quality of life.

### 2.2 Participants

Participants were selected using a snowballing technique carried out with the general population.

For eligibility to participate in the study, the following inclusion criteria must be met: (i) to have good reading comprehension in Spanish, (ii) to be of legal age, (iii) to have signed the informed consent form prior to participation.

The sample size consisted of 745 participants. The average age of the participants was 39.9 years old (SD = 16.99), and 67.1% were women (32.9% men). The rest of the sociodemographic data is shown in Table 1.

**Table 1:** Sociodemographic data of the participants.

Test *n* = 745				Retest *n* = 101	
Variable		*n*/Mean	%/SD	*n*/Mean	%/SD
Age		39.94	16.99	37.34	13.165
Gender	Man	245	32.9%	25	24.8%
	Woman	500	67.1%	76	75.2%
BMI	Under Weight	34	4.6%	5	5%
	Normal weight	377	50.6%	54	53.4%
	Overweight	224	30.1%	27	26.7%
	Obese	110	14.8%	15	14.9%
Place of residence	Rural	157	21.1%	24	23.8%
	Urban	588	78.9%	77	76.2%
Civil status	Single	241	32.3%	34	33.7%
	In a stable relationship	158	21.2%	26	25.7%
	Married	279	37.4%	35	34.7%
	Separated – Divorced	38	5.1%	5	5%
	Widowed	29	3.9%	1	1%
Individuals residing with the participant	Alone	66	8.9%	11	10.9%
	Partner	185	24.8%	24	23.8%
	Family	464	62.3%	62	61.4%
	Not family	30	4.0%	4	4%
Education level	No studies	31	4.2%	0	0%
	Primary level	76	10.2%	9	8.9%
	Secondary level	118	15.8%	15	14.9%
	High School Diploma/Vocational Training	288	38.7%	38	37.6%
	Undergraduate degree with a duration of 3, 4, or 5 years	154	20.7%	26	25.7%
	Master’s degree	66	8.9%	11	10.9%
	PhD	12	1.6%	2	2%
Working situation	Student	148	19.8%	26	25.7%
	Part-time employment	77	10.3%	14	13.9%
	Full-time employment	344	45.9%	46	45.5%
	Unemployed without pay	52	7.1%	5	5%
	Unemployed with pay	21	2.9%	3	3%
	Temporary leave of absence	24	3.3%	3	3%
	Permanent incapacity for work	12	1.7%	2	2%
	Retired	67	9%	2	2%
Monthly household income	No income	170	22.8%	24	23.8%
	Less than €500	76	10.2%	11	10.9%
	Between €501 and €1,000	154	20.7%	16	15.8%
	Between €1,001 and €1,500	196	26.3%	21	20.8%
	Between €1,501 and €2,000	126	16.9%	28	27.7%
	More than €2,000	23	3.1%	1	1%

### 2.3 Variables and instruments

#### 2.3.1 Sociodemographic data

An *ad hoc* questionnaire was used to collect the sociodemographic and clinical data. The following sociodemographic data were collected: age, gender, weight, height, nationality, place of residence, information on the individuals with whom the participant resides, education level, working situation, monthly household income, and civil status.

#### 2.3.2 Lifestyle

For the assessment of LS, the Spanish version of the Simple Lifestyle Indicator Questionnaire (SLIQ) was used (27). As previously mentioned, this questionnaire assesses LS among the general population. It consists of 12 items grouped into five dimensions: three for Diet, three for Exercise, three for Alcohol consumption, two for Tobacco use, and one for Life Stress. Each item in each dimension is scored separately on a scale from 0 to 5 for Diet, 0 to 4, 0–8, and 0–12 for Exercise, 0 to 2 for Tobacco use, 1 to 6 for Life Stress, and with no range for Alcohol consumption. However, these scores are grouped and assigned a single categorical score per dimension ranging from 0, 1, and 2. The criterion for assigning this score varies for each dimension. In the case of Diet, a 0 is assigned if the total direct score on the dimension is in the range of 0–5, a 1 if it is between 6–10 and a 2 if it is between 11–15. For the Exercise domain, a 0 is given if the individual only practices light exercise, a 1 if they exercise moderately and a 2 if they regularly engage in vigorous exercise. Regarding Alcohol consumption, a 0 is assigned if the alcohol raw score (number of drinks) is 14 or more, 1 if the raw score is in the range of 8–13 and 2 if it is between 0–7. As for Tobacco use, a 0 is given if the participant currently smokes, 1 is assigned they have ever smoked, and 2 they have never smoked. Finally, in the case of Life Stress, a 0 is assigned if life stress is 1 or 2, a 1 if life stress is 3 or 4, and a 2 if life stress is 5 or 6. The total score from the SLIQ questionnaire is the sum of the scores given in each of the five dimensions, ranging from 0 (“very unhealthy lifestyle”) to 10 (“very healthy lifestyle”). Overall, an individual’s LS is considered: “unhealthy” if they have an SLIQ score of between 0 and 4; “intermediate” if the score is between 5 and 7; and “healthy” if the person scores between 8 and 10 on the SLIQ questionnaire (27).

#### 2.3.3 Diet

The *PREDIMED* (40) questionnaire assesses adherence to the Mediterranean Diet (MD). This questionnaire consists of 14 items with a dichotomous response scale (0 or 1). The total PREDIMED score ranges between 0 and 14 points: higher scores are indicative of a higher level of adherence to the MD. One point is given when an individual’s responses to each item on the questionnaire are characteristic of the MD (for example, the use of olive oil as a main source of fat in cooking) (40).

#### 2.3.4 Physical activity

The short version of the International Physical Activity Questionnaire (IPAQ) questionnaire (41), consisting of seven items, assesses Physical Activity (PA) by recording walking time, sitting time, and the frequency, duration, and intensity of the exercise performed over the past 7 days. Specifically, this questionnaire assesses three properties of Physical Activity: intensity (light, moderate, or vigorous), frequency (days per week), and duration (time per day). According to the results, Physical Activity Level (PAL) is categorized as either “low,” “moderate,” “or high” (41).

#### 2.3.5 Depression, anxiety, and stress

The short-form version of the Depression, Anxiety, and Stress Scale (DASS-21) (42) assesses the presence and intensity of depressive symptoms, anxiety, and stress experienced over the past week. For this, each of the three scales contains seven items, assessed on a 4-point Likert scale ranging between 0 (“It has not happened to me”) and 3 (“It has happened to me a lot of the time, or most of the time”). The final score is calculated from the sum of the items of each scale, with a minimum total score of 0 points and a maximum of 21. A score above 11 indicates severe depression, a score above 8 indicates severe anxiety, and a score above 13 indicates severe stress (42).

#### 2.3.6 Health-related quality of life

The 12-Item Short Form Health Survey (SF-12) (43), validated with the Spanish population (44), is an instrument used to assess the quality of life of the general population. This instrument consists of 12 items and 8 dimensions: general health, physical functioning, physical role, emotional role, bodily pain, social functioning, mental health, and vitality. Using these 8 dimensions, two additional components are created: a Physical Health Component (PHC) and a Mental Health Component (MHC). The total score of the scale ranges from 0 (“worst health status”) to 100 (“best health status”).

### 2.4 Procedure

#### 2.4.1 Linguistic validation

In the process of translation-back translation and linguistic validation of the SLIQ questionnaire, a direct conceptual translation was performed from the original English version of the SLIQ into Spanish. Subsequently, a bilingual translator, unfamiliar with the original SLIQ instrument, translated the first Spanish version into English using a blind back-translation process. In the third step, a third unbiased translator compared the back-translated translation with the original version to check the linguistic equivalence while considering cultural differences. Here, minor changes were introduced such as the elimination of sports that are not practiced or that are practiced by a very small minority in Spain and therefore, are not well known (e.g., curling), the introduction of equivalences when some specific food products are referred (e.g., Raisin Bran), and specific changes regarding measures in the case of alcohol (e.g., oz. was replaced by ml). Finally, a committee consisting of four Spanish experts in LS assessment ensured the cultural and linguistic accuracy of the translated questionnaire. In terms of the instrument’s interpretability, a total of 20 cognitive interviews were conducted, in which the questionnaire’s pilot version was administered to the general population: 10 men and 10 women.

A psychologist trained in the assessment of health results collaborated in the interviews. Participants were asked to read the questionnaire aloud, fill it out, and indicate whether they understood or had any doubts about the meaning of the previous instructions, the items, and the alternative answers. When difficulties arose in reading comprehension, the interviewer helped the participant by clearly reading each item aloud. Finally, they were asked for an overall assessment of the specific instrument. There were no difficulties with understanding any of the items and all of them were adequately assessed by the interviewees. Therefore, none of the items were modified and the final version of the questionnaire was obtained in Spanish. The Spanish version of the SLIQ is included in Table 2.

**Table 2:** Spanish version of the SLIQ.

DIETA	Para contestar a las siguientes preguntas, piensa sobre tus hábitos alimentarios durante el último año. Indica con qué frecuencia consumes los siguientes alimentos. Por favor, para ello, ten en cuenta todas las comidas, aperitivos y alimentos que consumes fuera de casa.						
	Lechuga o ensalada de hojas verdes, con o sin otros vegetales	Menos de 1 a la semana	1 a la semana	2–3 veces a la semana	4–6 veces a la semana	1 al día	2 o más veces al día
	Fruta, incluida la fresca, enlatada o congelada, pero sin contar los zumos	Menos de 1 a la semana	1 a la semana	2–3 veces a la semana	4–6 veces a la semana	1 al día	2 o más veces al día
	Cereales ricos en fibra, como cereales de salvado con pasas, cereales ricos en fibra con fruta, copos de avena o pan elaborado con harina integral, multicereales o de centeno	Menos de 1 a la semana	1 a la semana	2–3 veces a la semana	4–6 veces a la semana	1 al día	2 o más veces al día
EJERCICIO	Para contestar a las siguientes preguntas, por favor, indica cuantas veces a la semana participas en las siguientes actividades durante al menos 30 minutos o más cada vez.						
	Ejercicio ligero como los siguientes: trabajo ligero de jardinería y pequeñas tareas domésticas (ej.: limpiar el polvo, barrer, pasar la aspiradora); pasear (ej.: pasear al perro); jugar a los bolos, pescar, realizar trabajos de carpintería, tocar un instrumento o desarrollar trabajo de voluntariado.	0 veces a la semana	1–3 veces a la semana	4–7 veces a la semana	8 o más veces a la semana		
	Ejercicio moderado como los siguientes: caminar a paso ligero, montar en bicicleta, patinar, nadar; realizar trabajos de jardinería (ej.: rastrillar, desmalezar, cavar); bailar, practicar Tai Chi o recibir clases de ejercicio moderado.	0 veces a la semana	1–3 veces a la semana	4–7 veces a la semana	8 o más veces a la semana		
	Ejercicio intenso como los siguientes: correr, montar en bicicleta, practicar esquí de fondo, nadar, hacer aerobic; realizar trabajos pesados de jardinería; practicar levantamiento de pesas o jugar al futbol, baloncesto u otros deportes de liga.	0 veces a la semana	1–3 veces a la semana	4–7 veces a la semana	8 o más veces a la semana		
CONSUMO DE ALCOHOL	Por favor, indica cuantas copas de los siguientes tipos de alcohol consumes normalmente en una semana:						
	Vino (copas 90–150 mL)						
	Cerveza (copas 300–330 mL o 1 botellín)						
	Bebidas espirituosas (copas 30–45 mL)						
CONSUMO DE TABACO	Por favor, indica tus hábitos tabáquicos a continuación:						
	¿Eres fumador/a?	Sí	No				
	Si no, ¿has fumado alguna vez?	Sí	No				
ESTRÉS	Para responder a esta pregunta, por favor rodea el número que mejor se corresponda con tu nivel de estrés en tu vida diaria	6 Nada estresante	5	4	3	2	1 Muy estresante

#### 2.4.2 Assessment procedure

The study was approved by the University of Alicante Research Ethics Committee (UA-2020-11-20) following the recommendations established in the Ethical Principles for Medical Research Involving Human Subjects (45). All participants were informed of the study objective and the confidentiality of the data collected, and prior to their participation, they signed an informed consent form, explaining that their authorization could be withdrawn or canceled at any time.

To administer of the questionnaires, a completely anonymous, and confidential online assessment protocol was created, including an information sheet about the study, informed consent for participation, and the distinct assessment instruments collected in the project. All of the questionnaires were administered in Spanish and all participants (*n* = 745) completed the full set of questionnaires included in the evaluation protocol: SLIQ, PREDIMED, IPAQ, DASS-21, and SF-12.

A non-probabilistic sample was used, selected using the “snowball sampling” technique. In this technique in the first phase of the study, participants who are representative of the target population are identified, requesting their collaboration to invite others with the same characteristics to participate in the study (46). This technique, widely used in biomedical and behavioral science research studies, has obtained good results as it permits access to a representative and relevant sample of the target population, while simultaneously reducing costs (in terms of both time and money) (46).

The degree of consistency and stability of the scores obtained from the SLIQ questionnaire was verified through a test–retest procedure. To this end, the Spanish version of the SLIQ was administered twice to a random selection of representative individuals of the obtained sample (*n* = 101) with a six-month time interval between the test and retest. This procedure permits the analysis of the degree of stability of the test over time and, thus, allowed for the quality of the data obtained to be verified.

### 2.5 Statistical analysis

Descriptive and frequency statistical analyses were used for data analysis. In addition, correlational analyses were conducted to establish associations between the variables analyzed, such as Pearson-type correlations and intraclass correlations. Based on the scores obtained in SLIQ, participants were divided according to the original cut-offs into three groups, depending on the quality of their lifestyle: unhealthy (*n* = 113), intermediate (*n* = 408), and healthy (*n* = 224). With the objective of establishing the differences between groups regarding depression, anxiety, stress and health-related quality of life (HRQoL), uni- or multivariate ANOVAs were performed. Statistical significance was established at *p* < 0.05. All analyses were carried out using the JASP statistical package (Version 0.16.3).

## 3 Results

### 3.1 Scores in each item and dimension of SLIQ

Table 3 shows the scores obtained for each item included in the SLIQ scale. In addition to the descriptive score of the five dimensions included in the SLIQ scale, Table 4 shows the score categorization including the percentage of participants within each category. The total questionnaire score is also categorized in this table.

**Table 3:** Descriptive scores of the items of the SLIQ scale.

Variables		Min	Max	Mean	SD
Diet	1. Lettuce or green leafy salad, with or without other vegetables	0	5	2.75	1.61
	2. Fruit, including fresh, canned, or frozen, but not including juices	0	5	3.55	1.41
	3. High-fiber cereals, such as Raisin Bran or Fruit and Fiber, cooked oatmeal, or whole-grain breads, such as whole wheat, rye, or pumpernickel	0	5	2.36	1.82
Exercise	4. Light exercise, such as the following: light gardening and light housework (e.g., dusting, sweeping, vacuuming); leisurely walking (e.g., walking your dog); bowling, fishing, carpentry, playing a musical instrument; volunteer work	0	4	2.54	1.02
	5. Moderate exercise, such as the following: brisk walking; bicycling, skating, swimming, curling; gardening (eg, raking, weeding, digging); dancing, Tai Chi, or moderate exercise classes	0	8	5.40	3.43
	6. Vigorous exercise, such as the following: running, bicycling, cross-country skiing, lap swimming, aerobics; heavy yard work; weight training; soccer, basketball, or other league sports	0	12	3.14	3.68
Alcohol consumption	7. Please indicate how many drinks of the following types of alcohol you consume in an average week: Wine	0	20	0.72	1.58
	8. Please indicate how many drinks of the following types of alcohol you consume in an average week: Beer	0	26	1.66	2.96
	9. Please indicate how many drinks of the following types of alcohol you consume in an average week: Spirits	0	10	0.38	1.13
Tobacco use	10. Smoking: Please indicate your smoking habits below: Are you a smoker?	0	0	0	0
	11. Smoking: Please indicate your smoking habits below: If not, did you ever smoke?	1	2	1.55	0.49
Life stress	12. To answer this question please circle the number which you feel best corresponds to the level of stress in your everyday life	1	6	3.45	1.35

**Table 4:** Descriptive scores of the categories of the SLIQ scale.

Variable	Categories	*n* (%)	Min	Max	Mean	SD
Diet	Poor	(142) (19.1%)	0	15	8.67	3.38
	Moderate	(339) (45.5%)				
	Good	(264) (35.4%)				
Exercise	Light	(182) (24.4%)	0	24	11.08	6.02
	Moderate	(234) (31.4%)				
	Vigorous	(329) (44.2%)				
Alcohol consumption	High	(23) (3.1%)	0	35	2.75	4.29
	Moderate	(45) (6%)				
	Low	(677) (90.9%)				
Tobacco use	Current smoker	(154) (20.7%)	0	2	1.23	0.76
	Former smoker	(267) (35.8%)				
	Non-smoker	(324) (43.5%)				
Life stress	Low	(197) (26.5%)	1	6	3.45	1.35
	Moderate	(369) (49.5%)				
	High	(179) (24%)				
Total healthy lifestyle	Unhealthy	(113) (15.2%)	1	10	6.44	1.78
	Intermediate	(408) (54.8%)				
	Healthy	(224) (30.1%)				

### 3.2 Convergent validity

Table 5 shows the correlations of the diet, exercise, and stress dimensions of the SLIQ questionnaire with their reference standards: the PREDIMED questionnaire for Diet, the IPAQ questionnaire for Exercise, and the Stress subscale of the DASS-21, respectively. In all cases, the correlations are significant.

**Table 5:** Analysis of convergent validity between the dimensions of the SLIQ and referent questionnaires.

	Mean (SD)	SLIQ-Diet	SLIQ-Exercise	SLIQ Life Stress	SLIQ_Total Healthy Lifestyle
PREDIMED	7.97 (2.20)	0.466**	0.158**	0.044	0.311**
IPAQ	1.17 (0.81)	0.181**	0.490**	−0.016	0.298**
DASS-21 Stress	5.93 (4.63)	−0.134**	−0.075*	−0.369**	−0.331**

* Correlation is significant at the 0.01 level (bilateral). ** Correlation is significant at the 0.05 level (bilateral).

### 3.3 Test–retest reliability

The intraclass coefficient correlation (ICC) for repeated measures in the SLIQ questionnaire was 0.836 (*p* < 0.001) for the total diet score, 0.921 (*p* < 0.001) for exercise, 0.978 (*p* < 0.001) for alcohol consumption, 1.000 for tobacco use, 0.825 (*p* < 0.001) for stress, and 0.923 (*p* < 0.001) for total healthy lifestyle score. According to the literature, this indicates good to excellent agreement.

The test–retest reliability for the different items of the scale ranged from 0.718 for fiber consumption to 1.000 for tobacco use item, respectively (Table 6).

**Table 6:** Intraclass correlation coefficients (ICC) between the first and second measurements of the SLIQ scale.

Variables	First test mean	Second test mean	ICC	95%CI		*P*
				Lower limit	Upper limit	
DIET score	8.56	7.54	0.836	0.770	0.902	<0.001
1. Lettuce or green leafy salad, with or without other vegetables	2.65	2.19	0.797	0.666	0.872	<0.001
2. Fruit, including fresh, canned, or frozen, but not including juices	3.41	3.21	0.774	0.665	0.848	<0.001
3. High-fiber cereals, such as Raisin Bran or Fruit and Fiber, cooked oatmeal, or whole-grain breads, such as whole wheat, rye, or pumpernickel	2.50	2.15	0.718	0.580	0.810	<0.001
EXERCISE score	10.38	10.61	0.921	0.883	0.947	<0.001
4. Light exercise, such as the following: light gardening and light housework (e.g., dusting, sweeping, vacuuming); leisurely walking (e.g., walking your dog); bowling, fishing, carpentry, playing a musical instrument; volunteer work	2.59	2.71	0.734	0.606	0.820	<0.001
5. Moderate exercise, such as the following: brisk walking; bicycling, skating, swimming, curling; gardening (e.g., raking, weeding, digging); dancing, Tai Chi, or moderate exercise classes	5.47	5.64	0.878	0.820	0.918	<0.001
6. Vigorous exercise, such as the following: running, bicycling, cross-country skiing, lap swimming, aerobics; heavy yard work; weight training; soccer, basketball, or other league sports	2.32	2.26	0.880	0.822	0.919	<0.001
ALCOHOL CONSUMPTION score	2.47	2.32	0.978	0.968	0.985	<0.001
7. Please indicate how many drinks of the following types of alcohol you consume in an average week: Wine	0.72	0.77	0.982	0.973	0.988	<0.001
8. Please indicate how many drinks of the following types of alcohol you consume in an average week: Beer	1.48	1.32	0.968	0.952	0.979	<0.001
9. Please indicate how many drinks of the following types of alcohol you consume in an average week: Spirits	0.27	0.23	0.960	0.941	0.973	<0.001
TOBACCO USE score	1.29	1.29	1.000	–	–	–
10. Smoking: Please indicate your smoking habits below: Are you a smoker?	0	0	–	–	–	–
11. Smoking: Please indicate your smoking habits below: If not, did you ever smoke?	1.55	1.55	1.000	–	–	–
LIFE STRESS score	3.11	3.16	0.825	0.740	0.882	<0.001
12. To answer this question, please circle the number you feel best corresponds to the level of stress in your everyday life	3.11	3.16	0.825	0.740	0.882	<0.001
TOTAL LIFESTYLE score	6.20	6.10	0.923	0.886	0.948	<0.001

### 3.4 Differences regarding levels of depression, anxiety, and HRQoL as a function of lifestyle

To identify the discrimination power of the Spanish version of the SLIQ on health outcomes, the differences in HRQoL, anxiety, depression and stress based on the level of adherence to a healthy LS were evaluated. Hence, participants were initially classified into three groups according to their scores on the SLIQ questionnaire for LS (“unhealthy,” “intermediate,” and “healthy”) (Table 7).

**Table 7:** Differences regarding levels of depression, anxiety, stress, and HRQoL as a function of lifestyle.

Variable	Mean (SD) Total sample (*n* = 745)	Categories	Mean	SD
SF12_Physical Functioning	86.17 (24.86)	Unhealthy	80.97	25.72
		Intermediate	85.60	25.44
		Healthy	89.84	22.83
SF12_Physical Role	73.62 (39.30)	Unhealthy	68.58	41.29
		Intermediate	72.92	39.75
		Healthy	77.46	37.20
SF12_Body Pain	78.83 (27.32)	Unhealthy	70.35	31.96
		Intermediate	78.25	27.32
		Healthy	84.15	23.47
SF12_General Health	53.62 (20.86)	Unhealthy	47.57	21.38
		Intermediate	51.65	19.64
		Healthy	60.27	21.19
SF12_Vitality	58.23 (25.27)	Unhealthy	49.38	27
		Intermediate	56.86	24.60
		Healthy	65.18	23.85
SF12_Social Functioning	86.61 (23.85)	Unhealthy	80.31	28.23
		Intermediate	87.56	22.78
		Healthy	88.06	22.94
SF12_Emotional Role	69.13 (42.91)	Unhealthy	57.08	46.22
		Intermediate	67.40	43.67
		Healthy	78.35	37.73
SF12_Mental Health	63.22 (19.75)	Unhealthy	56.11	22.01
		Intermediate	61.74	19.06
		Healthy	69.51	18.04
SF12_Physical Health Component	49.88 (10.25)	Unhealthy	48.25	10.36
		Intermediate	49.70	10.40
		Healthy	51.05	9.80
SF12_Mental Health Component	43.44 (12.80)	Unhealthy	39.10	14.12
		Intermediate	42.89	12.93
		Healthy	46.61	11
DASS21_Depression	4.25 (4.83)	Unhealthy	6.41	5.97
		Intermediate	4.40	4.79
		Healthy	2.88	3.68
DASS21_Anxiety	3.62 (4.09)	Unhealthy	5.33	4.43
		Intermediate	3.77	4.27
		Healthy	2.48	3.12
DASS21_Stress	5.93 (4.63)	Unhealthy	8.51	4.48
		Intermediate	6.10	4.75
		Healthy	4.30	3.79

In the case of HRQoL, differences between groups were found in physical functioning (PF) [*F*(2, 742) = 5.073, *p* = 0.006, η2 partial = 0.013], bodily pain (BP) [*F*(2, 742) = 10.017, *p* < 0.001, η2 partial = 0.026], general health (GH) [*F*(2, 742) = 18.790, *p* < 0.001, η2 partial = 0.048], vitality (VT) [*F*(2, 742) = 16.662, *p* < 0.001, η2 partial = 0. 043], social functioning (SF) [*F*(2, 742) = 4.726, *p* = 0.009, η2 partial = 0.013], emotional role (ER) [*F*(2, 742) = 10.202, *p* < 0.001, η2 partial = 0. 027], mental health (MH) [*F*(2, 742) = 20.888, *p* < 0.001, η2 partial = 0.053], and Mental Health Summary (MHS) Score [*F*(2, 742) = 14.238, *p* < 0.001, η2 partial = 0.037].

Differences between the three groups were revealed after the *post-hoc* analysis was performed, revealing differences between participants with high adherence to a healthy LS as compared to those with low adherence (*p* < 0.05). No significant differences were found in the analyses for physical role (PR) and the Physical Health Summary Score (*p* > 0.05).

In the case of anxiety, stress, and depression levels, differences between groups were found regarding the levels of depression [*F*(2, 742) = 21.563, *p* < 0.001, η2 partial = 0.055], anxiety [*F*(2, 742) = 19.827, *p* < 0.001, η2 partial = 0.051], and stress [*F*(2, 742) = 34.422, *p* < 0.001, η2 partial = 0.085]. In this case, the post-hoc analysis revealed significant differences between the three groups (*p* < 0.05) (Table 7).

## 4 Discussion

The aim of this study was to carry out the transcultural adaptation of the SLIQ questionnaire to Spain and validate it. This is a short and easy-to-apply instrument that provides a global lifestyle score, as well as specific scores for each of its components. After analyzing the data, the results appear to indicate that the Spanish version presents adequate psychometric properties to assess the LS of the general population. Regarding the translation process and the transcultural adaptation of the questionnaire, the results show an adequate equivalence between both the English and Spanish items, which apply to the Spanish context without the need for further modifications.

Regarding psychometric properties, to assess the convergent validity, associations between the SLIQ dimensions and different standardized referral questionnaires were analyzed. Statistically significant correlations were obtained from these analyses, which were similar to those obtained in the study by the authors of the original scale (6). Specifically, in the study by Godwin et al. (6), the correlations between the dimensions of diet and alcohol consumption from the SLIQ questionnaire and the scores from the Diet History Questionnaire were analyzed, obtaining *r* = 0.679 and 0.665, respectively (6). Regarding physical activity, a pedometer was used to measure the number of steps taken over 3 days and correlate it with the scores of the exercise dimension of the SLIQ questionnaire, obtaining *r* = 0.455 (6). Stress was another variable used by the authors of the original scale to assess convergent validity. It was measured using the Social Readjustment Rating Scale, obtaining a score of = −0.264 (6). Finally, with regard to concurrent validity, the eight-question scale on cardiovascular risk by Spencer et al. (47) was assessed, obtaining a reasonable correlation (*r* = 0.475) (47). In this study, the diet, exercise, and stress variables were correlated through the IPAQ and PREDIMED questionnaires and the stress dimension of the DASS-21 questionnaire, respectively. The correlation coefficients of diet, exercise, and stress analyzed in this study were 0.466, 0.490, and −0.369, respectively, thus displaying a significant relationship (*p* < 0.01). Although the size of the obtained correlation coefficients was smaller than expected and lower in comparison to the previous studies indicated above, the results demonstrate that the Spanish version of the SLIQ questionnaire may be a valid instrument for the assessment of different LS dimensions, having significant associations with scores obtained in other standardized instruments considered the gold standard for the assessment of the same variables.

Moreover, the SLIQ questionnaire has been shown to have good test–retest reliability. Test–retest reliability was determined by calculating the ICCs (48). The ICC results were obtained between the first and second measurements of the SLIQ questionnaire for the total score of each scale item; the total score of the five dimensions (Diet, Exercise, Alcohol consumption, Tobacco use, and Stress); and the total healthy lifestyle score. General guidelines for clinical research were used to establish the criterion to define the correlation strength, with a correlation below 0.25 indicating little or no agreement, a correlation between 0.25 and 0.50 indicating some agreement, a correlation between 0.50 and 0.75 indicating moderate to good agreement, and a correlation above 0.75 indicating good to excellent agreement (49). According to the previous correlation strength criterion, only two items (fiber consumption and light exercise) from the SLIQ questionnaire obtained a score of moderate to good with a score of 0.718 and 0.734, respectively. The rest of the items obtained a correlation above 0.75, confirming that the SLIQ questionnaire has a good level of test–retest reliability; and therefore, the temporal stability of the scores was shown to be adequate. Moreover, observing the sociodemographic data of participants who agreed to participate in the retest, an equivalence regarding the sociodemographic profile obtained in the first administration of the test is confirmed. This reinforces the stability and reliability of the test, regardless of the respondent’s sociodemographic profile.

Finally, to analyze whether the questionnaire would permit the identification of LS profiles that may discriminate the health status of the population, the differences regarding HRQoL, depression, anxiety, and stress were analyzed among individuals in the unhealthy, intermediate, and healthy lifestyle groups. Differences were found for the SF-12 questionnaire in the dimensions of physical functioning, bodily pain, general health, vitality, social functioning, emotional role, mental health, and the mental health summary score. Regarding the DASS-21 questionnaire, these differences were found in the anxiety, stress, and depression dimensions, suggesting that an unhealthy lifestyle is related to higher levels of anxiety, stress, and depression. Likewise, for the SF-12 questionnaire, with the exception of the dimensions of the physical role and the physical health summary score, the other dimensions showed significant differences between the groups. These results are in line with those from past studies, which found that individuals with lifestyles having more health risk factors obtained lower scores on health-related quality of life (50, 51). Among the lifestyle risk factors, special note should be made of alcohol consumption, tobacco use, stress, unhealthy diet, and lack of physical activity (52–55). Likewise, as in this study, the ASPREE work (56) revealed differences in quality of life depending on the population’s lifestyle, with those consuming less alcohol and leading a more active life obtaining higher scores on the physical and mental components, while smokers obtained worse results in the physical component, implying a poorer quality of life (56). Many studies show that regular physical activity and a healthy diet improve HRQoL and well-being (57, 58). However, alcohol consumption and tobacco use, as well as high stress levels, are related to a poorer quality of life, having a great negative impact on HRQoL (59–61). Likewise, a healthy LS can contribute to the prevention of health diseases, especially chronic and cardiovascular ones (23, 33, 35). As a result, the SLIQ questionnaire has been shown to be valid and reliable for identifying differences in LS and its relationship with health outcomes.

### 4.1 Limitations

The results of this study demonstrate the reliability and validity of the SLIQ questionnaire for its use in Spain. Although this represents an advance in the validation of short, valid, and reliable instruments for their use in different clinical and research contexts, some study limitations should be mentioned. Firstly, it is worth noting that the questionnaires were self-reported, which may imply certain comprehension biases and, therefore, could alter the results. Moreover, fewer participants agreed to take part in the retest, and other variables that may have influenced the results of the retest, such as health literacy, were not evaluated. Additionally, the possible differences in SLIQ results between participants from urban versus rural contexts have not been considered in the present study due to comprehension biases or difficulties in completing self-report measures. Future studies should use longitudinal methodologies with larger sample sizes to corroborate the results of this work.

## 5 Conclusion

It has been shown that LS is highly related to the population’s quality of life and well-being. It also serves as a protective factor against the development of different diseases. Therefore, most studies analyze health risk factors in an individualized manner, with fewer studies undertaking a global analysis, including diet, exercise, alcohol consumption, tobacco use, and stress, through short, valid, and effective instruments. The results obtained in this study show that the Spanish version of the SLIQ questionnaire is a reliable instrument for the assessment of the population’s lifestyle and, therefore, they show that its use by both clinics and researchers would provide fast, valid, and highly reliable results on the population’s LS.

## Data availability statement

The original contributions presented in the study are included in the article, further inquiries can be directed to the corresponding author.

## Ethics statement

The studies involving humans were approved by University of Alicante Research Ethics Committee (UA-2020-11-20). The studies were conducted in accordance with the local legislation and institutional requirements. The participants provided their written informed consent to participate in this study.

## Author contributions

EM-S, NR-R, and RF-C: conceptualization. NA-B and NR-R: methodology. EM-S and NA-B: formal analysis. EM-S: investigation. CA-B and VC-C: resources. EM-S, RF-C, NA-B, and VC-C: data curation. NR-R and RF-C: supervision. EM-S, NR-R, and VC-C: writing original draft preparation. All authors contributed significantly to the revision of the manuscript, provided scientific guidance, and read and approved the final manuscript.

## References

[1] Wilson DMC Ciliska D. Lifestyle assessment. Can Fam Physician. (1984) 30:1527–32.

[2] Mak YW Kao AHF Tam LWY Tse VWC Tse DTH Leung DYP. Health-promoting lifestyle and quality of life among Chinese nursing students. Prim Health Care Res Dev. (2018) 19:629–36. doi: 10.1017/S1463423618000208, PMID: 29623871 PMC6692834

[3] Nudelman G Yakubovich S. Patterns of health lifestyle behaviours: findings from a representative sample of Israel. BMC Public Health. (2022) 22:2099. doi: 10.1186/s12889-022-14535-5, PMID: 36384549 PMC9670447

[4] WHOQOL Group. The World Health Organization quality of life assessment (WHOQOL): position paper from the World Health Organization. Soc Sci Med. (1995) 41:1403–9. 8560308 10.1016/0277-9536(95)00112-k

[5] Bontrup C Taylor WR Fliesser M Visscher R Green T Wippert P-M. Low back pain and its relationship with sitting behaviour among sedentary office workers. Appl Ergon. (2019) 81:102894. doi: 10.1016/j.apergo.2019.102894, PMID: 31422243

[6] Godwin M Pike A Bethune C Kirby A Pike A. Concurrent and convergent validity of the simple lifestyle Indicator questionnaire. ISRN Fam Med. (2013) 2013:529645. doi: 10.5402/2013/529645, PMID: 24967324 PMC4041224

[7] Anderson AR Fowers BJ. Lifestyle behaviors, psychological distress, and well-being: a daily diary study. Soc Sci Med. (2020) 263:113263. doi: 10.1016/j.socscimed.2020.113263 32805573

[8] Balanzá-Martínez V Kapczinski F de Azevedo CT Atienza-Carbonell B Rosa AR Mota JC. The assessment of lifestyle changes during the COVID-19 pandemic using a multidimensional scale. Rev Psiquiatr Salud Ment. (2021) 14:16–26. doi: 10.1016/j.rpsm.2020.07.003, PMID: 38620670 PMC7456305

[9] Zhang C Lakens D Ijsselsteijn WA. Theory integration for lifestyle behavior change in the digital age: an adaptive decision-making framework. J Med Internet Res. (2021) 23:e17127. doi: 10.2196/17127, PMID: 33835036 PMC8065564

[10] Nari F Jeong W Jang BN Lee HJ Park E-C. Association between healthy lifestyle score changes and quality of life and health-related quality of life: a longitudinal analysis of south Korean panel data. BMJ Open. (2021) 11:e047933. doi: 10.1136/bmjopen-2020-047933, PMID: 34675011 PMC8532554

[11] Zheng X Xue Y Dong F Shi L Xiao S Zhang J. The association between health-promoting-lifestyles, and socioeconomic, family relationships, social support, health-related quality of life among older adults in China: a cross sectional study. Health Qual Life Outcomes. (2022) 20:64. doi: 10.1186/s12955-022-01968-0, PMID: 35443689 PMC9022255

[12] Solera-Sanchez A Adelantado-Renau M Moliner-Urdiales D Beltran-Valls MR. Health-related quality of life in adolescents: individual and combined impact of health-related behaviors (DADOS study). Qual Life Res. (2021) 30:1093–101. doi: 10.1007/s11136-020-02699-9, PMID: 33196960

[13] Zou S Feng G Li D Ge P Wang S Liu T. Lifestyles and health-related quality of life in Chinese people: a national family study. BMC Public Health. (2022) 22:2208. doi: 10.1186/s12889-022-14680-x, PMID: 36443710 PMC9706972

[14] Li Y Schoufour J Wang DD Dhana K Pan A Liu X. Healthy lifestyle and life expectancy free of cancer, cardiovascular disease, and type 2 diabetes: prospective cohort study. BMJ. (2020) 368:l6669. doi: 10.1136/bmj.l6669, PMID: 31915124 PMC7190036

[15] van Oort S Beulens JWJ van Ballegooijen AJ Burgess S Larsson SC. Cardiovascular risk factors and lifestyle behaviours in relation to longevity: a Mendelian randomization study. J Intern Med. (2021) 289:232–43. doi: 10.1111/joim.13196 33107078 PMC7894570

[16] Zhang Y-B Pan X-F Chen J Cao A Zhang Y-G Xia L. Combined lifestyle factors, incident cancer, and cancer mortality: a systematic review and meta-analysis of prospective cohort studies. Br J Cancer. (2020) 122:1085–93. doi: 10.1038/s41416-020-0741-x, PMID: 32037402 PMC7109112

[17] Botteri E Berstad P Sandin S Weiderpass E. Lifestyle changes and risk of cancer: experience from the Swedish women’s lifestyle and health cohort study. Acta Oncol. (2021) 60:827–34. doi: 10.1080/0284186X.2021.1919756, PMID: 33988490

[18] George EK Reddy PH. Can healthy diets, regular exercise, and better lifestyle delay the progression of dementia in elderly individuals? J Alzheimers Dis. (2019) 72:S37–58. doi: 10.3233/JAD-190232, PMID: 31227652

[19] Jeruszka-Bielak M Kollajtis-Dolowy A Santoro A Ostan R Berendsen AAM Jennings A. Are nutrition-related knowledge and attitudes reflected in lifestyle and health among elderly people? A study across five European countries. Front Physiol. (2018) 9:9. doi: 10.3389/fphys.2018.00994, PMID: 30108512 PMC6079245

[20] Kivipelto M Mangialasche F Ngandu T. Lifestyle interventions to prevent cognitive impairment, dementia and Alzheimer disease. Nat Rev Neurol. (2018) 14:653–66. doi: 10.1038/s41582-018-0070-3 30291317

[21] Rasmussen L Poulsen CW Kampmann U Smedegaard SB Ovesen PG Fuglsang J. Diet and healthy lifestyle in the management of gestational diabetes mellitus. Nutrients. (2020) 12:3050. doi: 10.3390/nu12103050, PMID: 33036170 PMC7599681

[22] Wahl D Solon-Biet SM Cogger VC Fontana L Simpson SJ Le Couteur DG. Aging, lifestyle and dementia. Neurobiol Dis. (2019) 130:104481. doi: 10.1016/j.nbd.2019.104481 31136814

[23] Patnode CD Evans CV Senger CA Redmond N Lin JS. Behavioral counseling to promote a healthful diet and physical activity for cardiovascular disease prevention in adults without known cardiovascular disease risk factors: updated evidence report and systematic review for the US preventive services task force. JAMA. (2017) 318:175–93. doi: 10.1001/jama.2017.3303 28697259 PMC13292382

[24] Lee SM Kim S Jeong JH Hong CH Park YK Na HR. Impact of a multidomain lifestyle intervention on white matter integrity: the SUPERBRAIN exploratory sub-study. Front Aging Neurosci. (2023) 15:1242295. doi: 10.3389/fnagi.2023.1242295, PMID: 37799622 PMC10548201

[25] Ding Z Leung P-Y Lee T Chan AS. Effectiveness of lifestyle medicine on cognitive functions in mild cognitive impairments and dementia: a systematic review on randomized controlled trials. Ageing Res Rev. (2023) 86:101886. doi: 10.1016/j.arr.2023.101886, PMID: 36806378

[26] Friedman SM. Lifestyle (medicine) and healthy aging. Clin Geriatr Med. (2020) 36:645–53. doi: 10.1016/j.cger.2020.06.007 33010900

[27] Godwin M Streight S Dyachuk E van den Hooven C Ploemacher J Seguin R. Testing the simple lifestyle Indicator questionnaire. Can Fam Physician. (2008) 54:76–7. 18208960 PMC2293321

[28] Reis F Sá-Moura B Guardado D Couceiro P Catarino L Mota-Pinto A. Development of a healthy lifestyle assessment toolkit for the general public. Front Med. (2019) 6:134. doi: 10.3389/fmed.2019.00134, PMID: 31316985 PMC6610478

[29] Pill R Stott NCH. Development of a measure of potential health behaviour: a salience of lifestyle index. Soc Sci Med. (1987) 24:125–34. doi: 10.1016/0277-9536(87)90245-0, PMID: 3563555

[30] Downes L. Motivators and barriers of a healthy lifestyle scale: development and psychometric characteristics. J Nurs Meas. (2008) 16:3–15. doi: 10.1891/1061-3749.16.1.3, PMID: 18578106

[31] Darviri C Alexopoulos EC Artemiadis AK Tigani X Kraniotou C Darvyri P. The healthy lifestyle and personal control questionnaire (HLPCQ): a novel tool for assessing self-empowerment through a constellation of daily activities. BMC Public Health. (2014) 14:995. doi: 10.1186/1471-2458-14-995, PMID: 25253039 PMC4192765

[32] Znazen H Slimani M Bragazzi NL Tod D. The relationship between cognitive function, lifestyle Behaviours and perception of stress during the COVID-19 induced confinement: insights from correlational and mediation analyses. Int J Environ Res Public Health. (2021) 18:3194. doi: 10.3390/ijerph18063194, PMID: 33808777 PMC8003540

[33] Bonekamp NE Visseren FLJ Cramer MJ Dorresteijn JAN van der Meer MG Ruigrok YM. Long-term lifestyle change and risk of mortality and type 2 diabetes in patients with cardiovascular disease. Eur J Prev Cardiol. (2023). doi: 10.1093/eurjpc/zwad316, PMID: 37774501

[34] Valenzuela PL Santos-Lozano A Saco-Ledo G Castillo-García A Lucia A. Obesity, cardiovascular risk, and lifestyle: cross-sectional and prospective analyses in a nationwide Spanish cohort. Eur J Prev Cardiol. (2023) 30:1493–501. doi: 10.1093/eurjpc/zwad204 37317985

[35] Xie H Li J Zhu X Li J Yin J Ma T. Association between healthy lifestyle and the occurrence of cardiometabolic multimorbidity in hypertensive patients: a prospective cohort study of UK biobank. Cardiovasc Diabetol. (2022) 21:199. doi: 10.1186/s12933-022-01632-3, PMID: 36183084 PMC9526960

[36] Ben Ayed H Yaich S Ben Jemaa M Ben Hmida M Trigui M Jedidi J. Lifestyle behaviors and mental health in medical students. J Public Ment Health. (2018) 17:210–7. doi: 10.1108/JPMH-07-2018-0039

[37] Hanawi SA Saat NZM Zulkafly M Hazlenah H Taibukahn NH Yoganathan D. Impact of a healthy lifestyle on the psychological well-being of university students. (2020) 9:1–17.

[38] Carretero-Dios H Pérez C. Standards for the development and review of instrumental studies: considerations about test selection in psychological research. Int J Clin Health Psychol. (2007) 7:863–82.

[39] García-Celay IM León OG. Sistema de clasificación del método en los informes de investigación en Psicología. Int J Clin Health Psychol. (2005) 5:115–27.

[40] Schröder H Fitó M Estruch R Martínez-González MA Corella D Salas Salvadó J. A short screener is valid for assessing Mediterranean diet adherence among older Spanish men and women. J Nutr. (2011) 141:1140–5. doi: 10.3945/jn.110.135566, PMID: 21508208

[41] Craig CL Marshall AL Sjostrom M Bauman AE Ainsworth BE Pratt M. International physical activity questionnaire: 12-country reliability and validity. Med Sci Sports Exerc. (2003) 35:1381–95. doi: 10.1249/01.MSS.0000078924.61453.FB, PMID: 12900694

[42] Lovibond PF Lovibond SH. The structure of negative emotional states: comparison of the depression anxiety stress scales (DASS) with the Beck depression and anxiety inventories. Behav Res Ther. (1995) 33:335–43. doi: 10.1016/0005-7967(94)00075-U, PMID: 7726811

[43] Ware JE Kosinski M Keller SD. A 12-item short-form health survey: construction of scales and preliminary tests of reliability and validity. Med Care. (1996) 34:220–33. doi: 10.1097/00005650-199603000-00003 8628042

[44] Vilagut G María Valderas J Ferrer M Garin O López-García E Alonso J. Interpretación de los cuestionarios de salud SF-36 y SF-12 en España: componentes físico y mental. Med Clínica. (2008) 130:726–35. doi: 10.1157/13121076, PMID: 18570798

[45] World Medical Association. World Medical Association Declaration of Helsinki: Ethical Principles for Medical Research Involving Human Subjects. JAMA. (2013) 310:2191–4. doi: 10.1001/jama.2013.281053 24141714

[46] Johnson TP. Snowball sampling: introduction John Wiley & Sons, Ltd Statistics Reference Online. USA (2014).

[47] Spencer CA Jamrozik K Norman PE Lawrence-Brown M. A simple lifestyle score predicts survival in healthy elderly men. Prev Med. (2005) 40:712–7. doi: 10.1016/j.ypmed.2004.09.012, PMID: 15850869

[48] Cicchetti DV. Guidelines, criteria, and rules of thumb for evaluating normed and standardized assessment instruments in psychology. Psychol Assess. (1994) 6:284–90. doi: 10.1037/1040-3590.6.4.284

[49] Portney LG Watkins MP. Foundations of clinical research: applications to practice. Surv Ophthalmol. (2002) 47:598. doi: 10.1016/S0039-6257(02)00362-4

[50] Vilchez-Chavez AF Bernal Altamirano E Morales-García WC Sairitupa-Sanchez L Morales-García SB Saintila J. Healthy habits factors and stress associated with health-related quality of life in a Peruvian adult population: a cross-sectional study. J Multidiscip Healthc. (2023) 16:2691–700. doi: 10.2147/JMDH.S412962, PMID: 37720270 PMC10505026

[51] Vajdi M Farhangi MA. A systematic review of the association between dietary patterns and health-related quality of life. Health Qual Life Outcomes. (2020) 18:337. doi: 10.1186/s12955-020-01581-z, PMID: 33046091 PMC7552532

[52] Aras YG Tunç A Güngen BD Güngen AC Aydemir Y Demiyürek BE. The effects of depression, anxiety and sleep disturbances on cognitive impairment in patients with chronic obstructive pulmonary disease. Cogn Neurodyn. (2017) 11:565–71. doi: 10.1007/s11571-017-9449-x, PMID: 29147148 PMC5670087

[53] Li Y Pan A Wang DD Liu X Dhana K Franco OH. Impact of healthy lifestyle factors on life expectancies in the US population. Circulation. (2018) 138:345–55. doi: 10.1161/CIRCULATIONAHA.117.032047, PMID: 29712712 PMC6207481

[54] Loef M Walach H. The combined effects of healthy lifestyle behaviors on all cause mortality: a systematic review and meta-analysis. Prev Med. (2012) 55:163–70. doi: 10.1016/j.ypmed.2012.06.017, PMID: 22735042

[55] Zaman R Hankir A Jemni M. Lifestyle factors and mental health. Psychiatr Danub. (2019) 31:217–20. PMID: 31488729

[56] Stocks NP González-Chica DA Woods RL Lockery JE Wolfe RSJ Murray AM. Quality of life for 19,114 participants in the ASPREE (ASPirin in reducing events in the elderly) study and their association with sociodemographic and modifiable lifestyle risk factors. Qual Life Res Int J Qual Life Asp Treat Care Rehabil. (2019) 28:935–46. doi: 10.1007/s11136-018-2040-z, PMID: 30411180 PMC6924574

[57] Bonaccio M Di Castelnuovo A Bonanni A Costanzo S De Lucia F Pounis G. Adherence to a Mediterranean diet is associated with a better health-related quality of life: a possible role of high dietary antioxidant content. BMJ Open. (2013) 3:e003003. doi: 10.1136/bmjopen-2013-003003, PMID: 23943771 PMC3752056

[58] Olivares PR Gusi N Prieto J Hernandez-Mocholi MA. Fitness and health-related quality of life dimensions in community-dwelling middle aged and older adults. Health Qual Life Outcomes. (2011) 9:117. doi: 10.1186/1477-7525-9-117, PMID: 22192520 PMC3286398

[59] Coste J Quinquis L D’Almeida S Audureau E. Smoking and health-related quality of life in the general population. Independent relationships and large differences according to patterns and quantity of smoking and to gender. PLoS One. (2014) 9:e91562. doi: 10.1371/journal.pone.0091562, PMID: 24637739 PMC3956698

[60] Daeppen J-B Faouzi M Sanchez N Rahhali N Bineau S Bertholet N. Quality of life depends on the drinking pattern in alcohol-dependent patients. Alcohol Alcohol Oxf Oxfs. (2014) 49:457–65. doi: 10.1093/alcalc/agu027, PMID: 24863264

[61] de Frias CM Whyne E. Stress on health-related quality of life in older adults: the protective nature of mindfulness. Aging Ment Health. (2015) 19:201–6. doi: 10.1080/13607863.2014.924090, PMID: 24940847 PMC4299552

